# The role of shuntography in diagnosis of mechanic complications after implantation of ventriculoperitoneal shunts in patients with idiopathic normal pressure hydrocephalus: a retrospective clinical evaluation

**DOI:** 10.1007/s00234-021-02834-4

**Published:** 2021-11-26

**Authors:** Sergej Rot, Leonie Goelz, Holger Arndt, Pawel Gutowski, Ullrich Meier, Johannes Lemcke

**Affiliations:** 1 Department of Neurosurgery, Unfallkrankenhaus Berlin, Warener Str. 7, 12683 Berlin, Germany; 2 Institute of Radiology and Neuroradiology, Unfallkrankenhaus Berlin, Warener Str. 7, 12683 Berlin, Germany; 3 Department of Neurosurgery, Brodno Masovian Hospital, Warsaw Postgraduate Medical Centre, Warsaw, Poland

**Keywords:** Shuntography, Idiopathic normal-pressure hydrocephalus, Over-and underdrainage, Kiefer score

## Abstract

**Background:**

Mechanical obstruction of ventriculoperitoneal shunt (VPS) during the first year after shunt implantation is a common complication and is widely described in the literature. In this paper, we evaluated the suitability of the shuntography for the diagnosis of mechanical complications of the VPS in patients with idiopathic normal pressure hydrocephalus (iNPH).

**Methods:**

We retrospectively identified 49 patients with pathologic shuntography over of a period of 20 years in our hospital. The percentage of procedure-associated complications was determined.

**Results:**

Ninety-eight percent (*n* = 48) of the patients who underwent shuntography showed clinical and radiographic signs of underdrainage prior to examination. Shuntography revealed mechanical complications of the VP shunt in 37% (*n* = 18) as a cause of clinical deterioration and following revision operation. During shuntography, mechanical obstruction was discovered in 78% (*n* = 14) and disconnection of shunt components in 22% (*n* = 4). In the obstruction group, in 50% (*n* = 7) the closure was detected in the ventricular catheter, in 29% (*n* = 4) in the distal catheter of the VPS, and in 21% (*n* = 3) in both sides of the VPS. In the case of an inconspicuous shuntography (63%, *n* = 31), the patients received symptomatic therapy (32%, *n* = 10) or re-adjustment of the valve setting (68%, *n* = 21). Fifty-seven percent of the patients who underwent surgical treatment improved clinically by at least one point according to the Kiefer score.

**Conclusion:**

Shuntography can produce valuable clinical information uncovering mechanic complications after implantation VPS in patients with idiopathic normal-pressure hydrocephalus. Patients with mechanical complications of their VPS needed revision surgery and showed clinical benefit after treatment.

## Background

Idiopathic normal pressure hydrocephalus (iNPH) of the elderly remains an enigmatic disease. Internal cerebrospinal fluid (CSF) diversion is established as the standard therapy, but in cases of poor outcome after ventriculoperitoneal shunting (VPS) or secondary deterioration, it can be difficult to differentiate between non-responders and patients with mechanical shunt dysfunction [1, 14]. Thus, every neurosurgeon is familiar with the trade-off between wait and see, non-invasive diagnostics, invasive functional diagnostics, or even revision surgery. Sufficient diversion of CSF is the main task of the VPS in the management of the disease “hydrocephalus.” In well-functioning VPS therapies, the clinical symptoms of iNPH such as gait disturbance, short-term memory disorder, and incontinence (Hakim triad), but also headaches and dizziness, are potentially reversible [5, 11, 16]. Several types of shunts are available but those with abdominal lead are implanted most often. Typically, the VPS consists of three components: the proximal catheter (ventricular catheter), shunt valve, and the distal catheter (abdominal drainage tube). All of these segments of the shunt may interfere with the viability of the VPS due to obstruction or disconnection which can lead to a complete failure. As a result, under-drainage of CSF may occur in the case of obstruction with clinical deterioration of gait disturbance or incontinence, or over drainage in disconnection with the leading symptom of headache [21]. Several studies have shown that more than 30% of patients with normal pressure hydrocephalus develop a mechanical complication due to obstruction of the VPS during the first year after shunt implantation [26, 27]. Only half of the newly implanted VPS function adequately during the second year, and less than one third works until the tenth year without revision. Clinical symptoms as well as assessments of internal and external CSF spaces on cranial imaging can signal shunt dysfunctions. However, these signs are not specific for mechanical complications in the course of the VPS therapy. In cases when the cause of a patient’s clinical worsening is not evident, functional contrast-enhanced shuntography may be helpful [30]. Mirfakhraee first described contrast medium-assisted shuntography in 1985 [19]. However, literature on experiences with shuntography is scant and mainly consists of studies with small case numbers. There are no guidelines for shuntography nor protocols for the procedure or consensus on image interpretation, which severely limits its’ clinical validity. This retrospective cohort study aimed to analyze contrast-enhanced shuntography by a quantitative method and to evaluate its’ clinical value for decision-making in patients with suspected VPS dysfunction. In addition, this study elaborates on a workflow for indicating, conducting, and interpreting shuntographies.

## Materials and methods

**Fig. 1:**
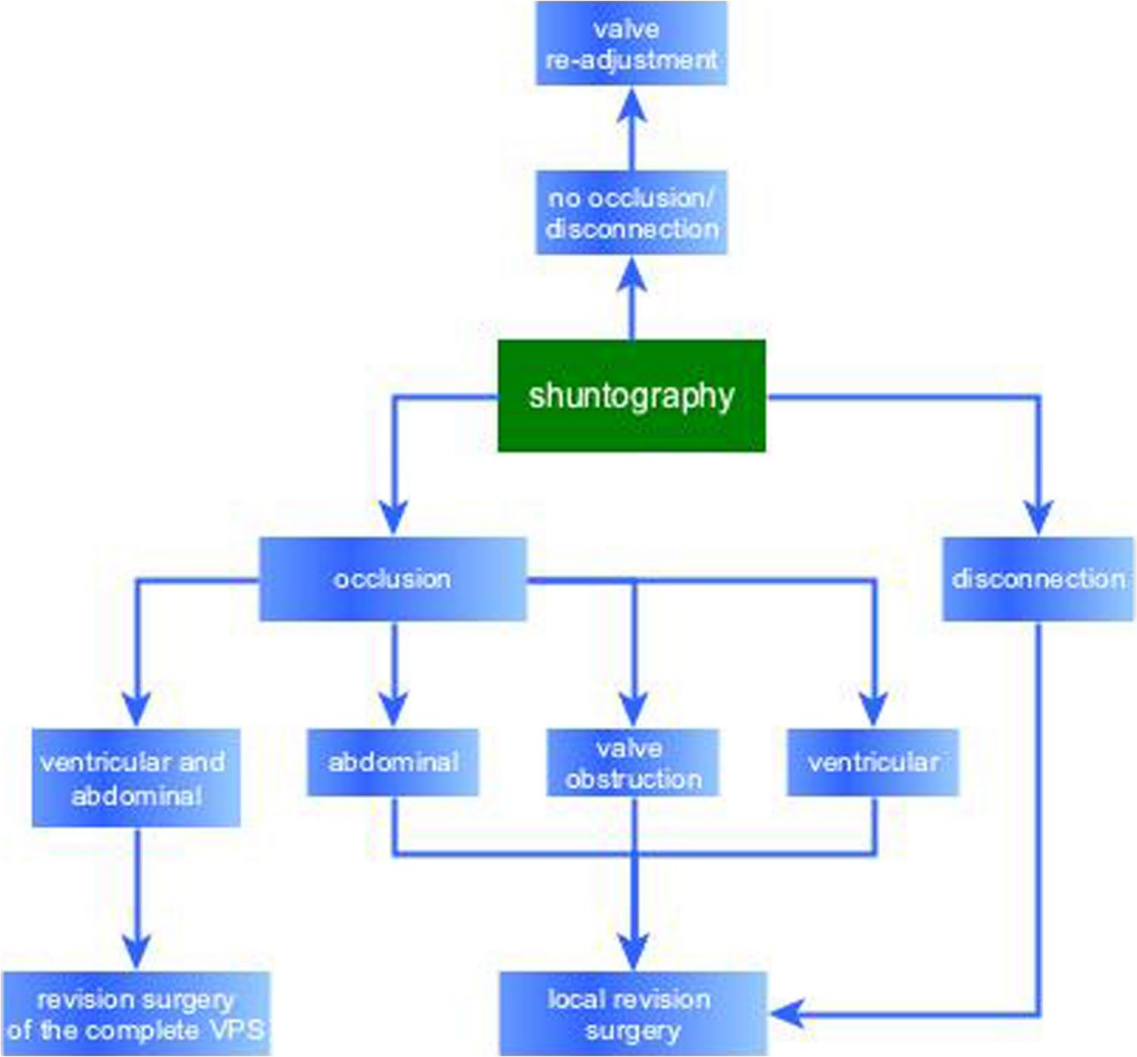
Flow diagram of decision processes after shuntography. Local revision surgery is the surgical revision of only one component of the VPS: ventricular, or abdominal catheter or valve

This retrospective cohort study included a total of 49 patients with iNPH who underwent ventriculoperitoneal shunting in an urban center of maximum care between 2000 and 2020. All patients showed secondary clinical worsening suspicious of mechanical complications of the VPS. Clinical symptoms of the patients, change of ventricular width on follow-up cranial imaging, and obstruction or disconnection of the VPS documented by shuntography were analyzed. The need for revision operations or for changes of the valve-pressure settings was also recorded (Fig. 1). Kiefer score was used as a good clinical outcome parameter and was recorded before shuntography and subsequent specific treatment [13].

### Protocol of conducting shuntography

Prior to shuntography in suspected mechanical complications of the VPS, the patient is hospitalized and a blood sample is drawn to exclude coagulopathies and relevant infections. A cranial CT scan is performed to evaluate ventricular width pre-interventionally. Additionally, a plain radiograph of the cranium, of the thorax, and the abdomen including the valve target image is acquired in order to detect possible disconnection in the shunt path. Just above the burr-hole reservoir, the hair is shaved to enable adequate soaking of the skin with a 7.5% iodinated solution (Braunovidon®) approximately 20 min before the procedure for local disinfection. The examination takes place in an angiography suit in supine position. At first fluoroscopy images of shunt system from the ventricular catheter to the retroauricular region which harbors the valve and gravitational unit in posterior-anterior projection. After removal of the swab, the burr-hole reservoir is covered with an incision drape and the skin is disinfected again. Then, the silicone membrane of the burr-hole reservoir is punctured with an 18 G butterfly needle and CSF is aspirated to test the patency of the ventricular catheter. Local anesthesia is not necessary, as patients tolerate the puncture of the borehole reservoir very well. In some cases, local anesthesia with EMLA plaster is used. If CSF can be aspirated, a quantity of approx. 10 ml is sent out for microbiological and chemical examination. Afterwards, a syringe with 10 ml of contrast medium for intrathecal application (Imeron M, containing Iodine) is connected to the butterfly and the needle is positioned into the direction of the ventricular catheter. Under fluoroscopy, a small amount (about 1–2 ml) of contrast medium is applied to visualize the patency of the ventricular catheter and to exclude a subgaleal leakage. The injection jet of the contrast medium through the openings at the sides and tip of the ventricular catheter in the direction of the lateral ventricles can be described. Then, the needle is diverted towards the valve and distal tube to trigger a distal flow towards the valve. The distal flow can be supported by changing the patient into a 30° tilted position. Only small bolus of the contrast medium (about 1 ml) is injected to see if the contrast medium column reaches the valve and then the gravitational unit. If the valve and gravitational unit are contrasted, the drainage of the contrast medium through the distal catheter is followed under fluoroscopy in a posterior-anterior projection. During fluoroscopy, the system is also checked for continuity to the abdomen. Here the tip of the distal catheter is magnified to observe a corresponding jet phenomenon of the contrast medium. In obese patients and meteorism, it can be challenging to visualize the contrast medium at the distal end of the catheter which can be overcome by increasing the radiation dose and contrast media bolus. Once the total of 10 ml contrast agent is applied, isotonic saline solution bolus is used to push the contrast medium column distally. At last, the amount of contrast agent and saline solution applied is aspirated and discarded. The needle is removed and the puncture site is covered with a sterile band-aid. After the examination, the patient stays in bed in 30° elevated upper body position for at least half an hour.

### Experimental shuntography in a patient model

**Fig. 2:**
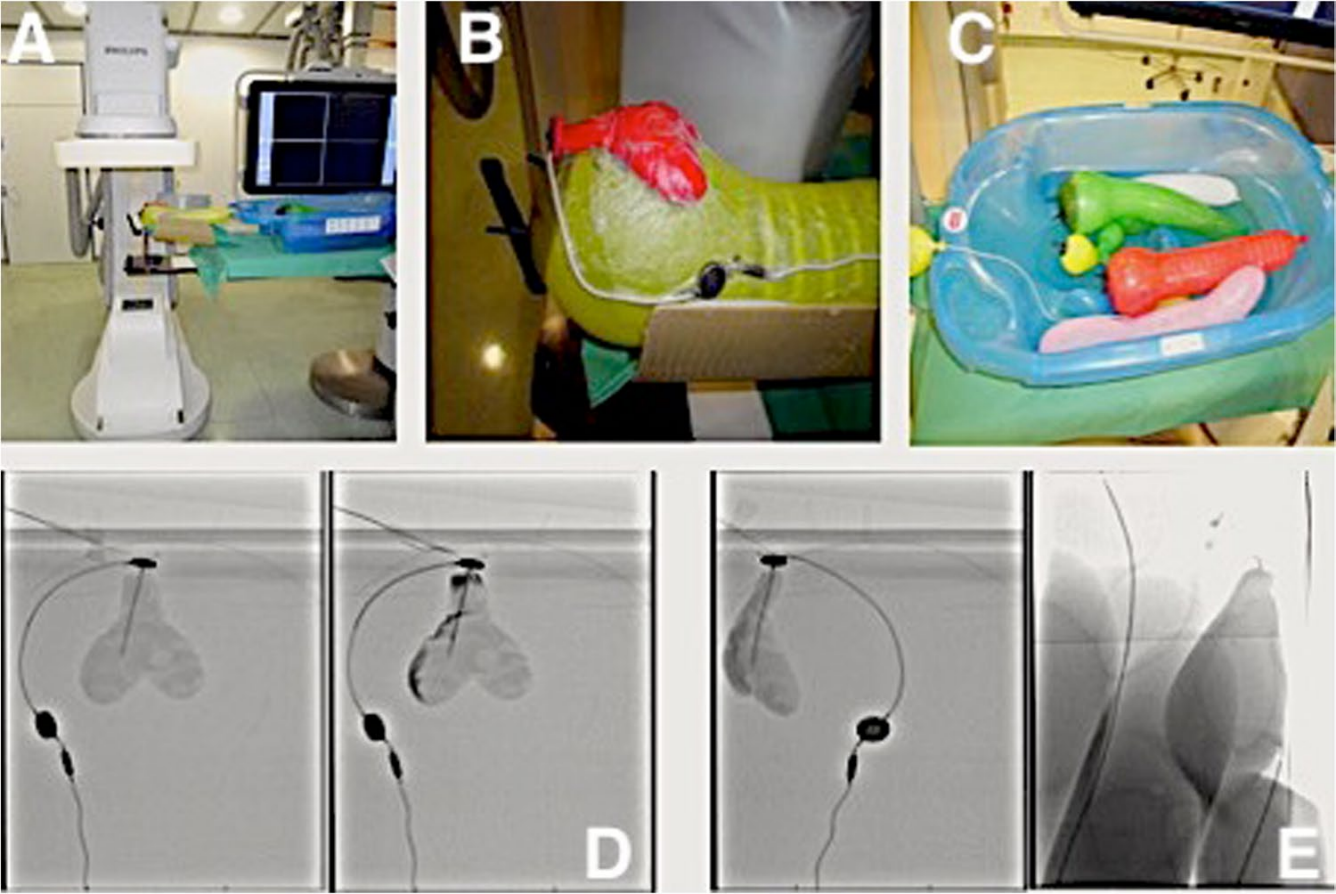
Experimental model of a shuntography. **A** Experimental setup, the model is placed on angiography stage. **B** Yellow balloon: simulation of the head and neck region. Red, heart-shaped balloon: simulation of the ventricular system. The balloon is filled with water and has an inserted ventricular catheter. **C** Blue children's bathtub is half filled with water: simulation of the abdomen. Color balloons: simulation of the intestinal loops. The distal catheter is lowered into the bath tub under the water filled balloons. **D** After injection of Imeron M radiographic image shows contrasting of the ventricular system. **E** Radiographic image shows distal flow of the contrast medium along the distal catheter

An experimental patient model was established to determine the minimal amount of contrast medium needed to prevent an increase in intracranial pressure during shuntography (Fig. 2A). The experimental setup consists of color balloons which simulate a head and neck region, ventricular system, and intestinal loops of a patient (Fig. 2A–C). Again, fluoroscopy images the ventricular catheter, the simulated ventricular system, and the valve with the gravitational unit in posterior-anterior projection (Fig. 2D). The burr-hole reservoir is punctured and the contrast medium is applied into the ventricular system until the distal flow of the contrast medium towards the valve the contrast medium the contrast medium column can be tracked to the tip of the distal catheter until the abdominal contrast medium jet becomes visible (Fig. 2E). In summary, experimental simulation of shuntography showed it required only 2–3 ml of contrast medium for and adequate visualization of a VPS. In the clinical setting, 3 ml marks the amount of contrast medium required under ideal conditions. According to our experience in the majority of cases, 5 ml of contrast medium is needed but as described above shuntography in obese patients can require up to 10 ml of contrast medium.

### Clinical case examples

**Fig. 3:**
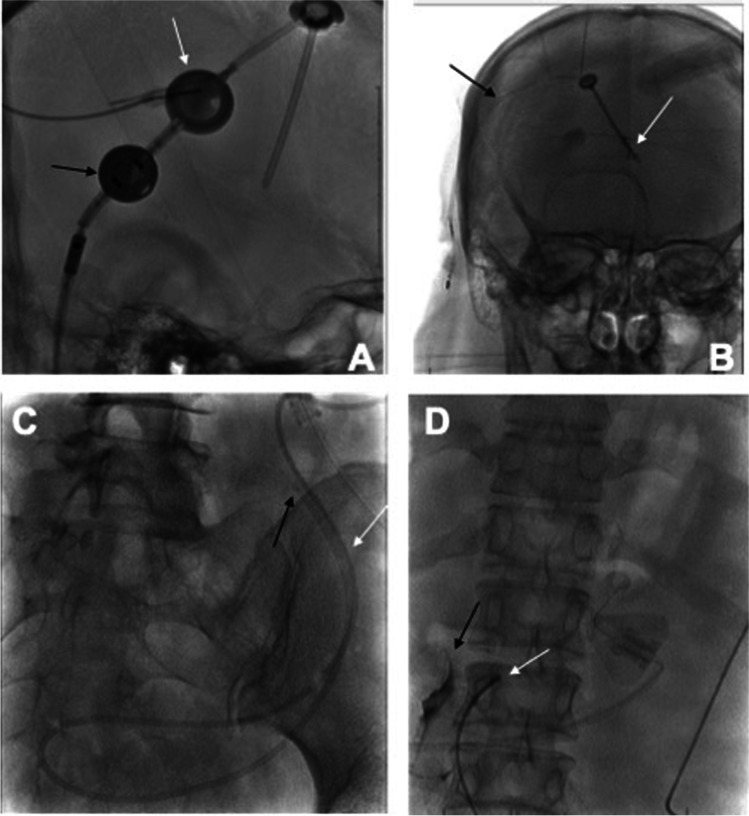
Clinical examples of radiographic imaging of shunt dysfunction (description in Table 1)

Table 1 shows the examples of NPH patients with clinical suspicion of a mechanical complication in the VPS, which can be visualized by a shuntogram (Fig. 3).

**Table 1:** Shuntographies of patients with shunt dysfunction

X-ray picture	Pathology designation	Description
A	Obstruction of proximal catheter	White arrow: butterfly needle placed in the prechamber, proximal catheter and ventricular catheter show typical double contour as a sign of passage disruption of the contrast medium; Black arrow: medium contrasted proGAV and gravitational unit including a visible part of distal catheter
B	Obstruction of the valve (proGAV/gravitational unit)	Black arrow: typical double contour of the connecting catheter between prechamber and Codmann Hackim valve as a sign of passage disruption of the contrast medium; White arrow: free passage of the contrast medium along the perforations of the ventricular catheter with particularly visible contrast medium in the right ventricle
C	Obstruction of abdominal catheter	White arrow: passage break of the contrast medium; Black arrow: no release of contrast medium at the distal end of the catheter, typical double contour of the non-contrasted catheter
D	A non-obstructed abdominal catheter (normal finding)	White arrow: release of the contrast medium from the tip of the abdominal catheter Black arrow: spread of the contrast medium along the intestinal loops

### Statistics

Numerical variables were expressed as mean ± standard deviation and categorical variables as percentages using Microsoft Excel for Mac (Microsoft Corp.).

## Results

Around 80 patients with NPH are treated in our clinic every year. Between 2000 and 2020, 49 patients with NPH underwent shuntography in our hospital. The median age of 53.1% male and 46.9% female patients was 66.2 ± 17.1 years (median, 70 years, age range, 54–93 years). The following valve types were used in patients, selected for the present study: proGAV, GAV, dual-switch, NMT (Miethke), Codmann-Hackim valve, OSV II (Integra). Ninety-eight percent (*n* = 48) of patients showed symptoms of under-drainage and 2% of them (*n* = 1) symptoms of over-drainage clinically and radiographically. Shuntography revealed mechanical complications of the VP shunt in 37% (*n* = 18) of cases as a cause of clinical deterioration. The cause of dysfunction in the shunt system was either obstruction or disconnection. Disconnection of the shunt system in the patient group with mechanical complications (*n* = 18) was discovered in 22% (*n* = 4). The radiological evaluations of shuntographies in patients who were advised to undergo revision surgery due to obstruction or disconnection of VPS were confirmed intraoperatively. Radiological evaluations were correct in all cases. Obstruction of the VPS was discovered in 78% (*n* = 14) of all mechanical complications (*n* = 18) and occurred at all parts of the VPS. In 50% (*n* = 7) of the cases, obstruction was detected in the ventricular catheter and in 29% (*n* = 4) in the distal catheter of the VPS. In 21% (*n* = 3) of cases, obstruction was found both in the ventricular and distal catheter of the VPS. A typical localized site or cause for obstruction of the shunt could not be determined in this study.

In the case of an inconspicuous shuntography (63%, *n* = 31) meaning in the case of evidence of neither obstruction nor disconnection of the VPS, the patients either received symptomatic therapy (32%, *n* = 10) or re-adjustment of the valve setting (68%, *n* = 21). The adaptation of the valve setting was patient-specific: in patients with headaches and rather narrow lateral ventricles, opening pressure of the low-pressure valve was increased; in patients with deterioration of gait disturbance expansion of internal CSF spaces, opening pressure was decreased.

**Fig. 4:**
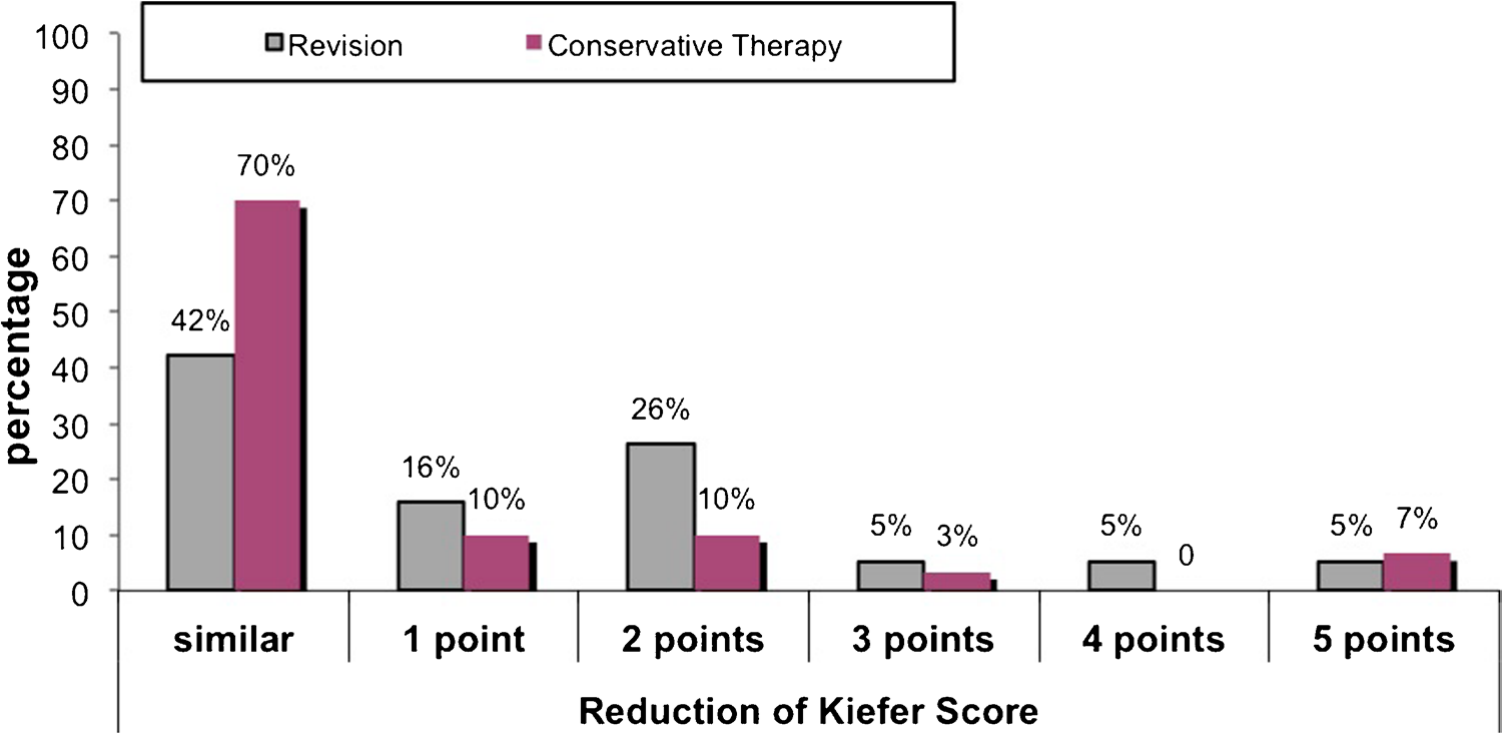
Clinical outcome after conservative and surgical treatment

Symptomatic therapy consisted of medication against headaches and dizziness as well as physiotherapeutic co-treatment to improve gait disturbance. Three months after surgical and conservative therapy, patients were followed up in the neurosurgical department. The Kiefer score served primary parameter for clinical evaluation of the patients. More than half of the patients (57%) improved clinically after revision surgery (reduction in Kiefer score by at least one point, Fig. 4, gray bars). In the conservative group, clinical symptoms were unchanged in 70% of the cases (Fig. 4, pink bars). An improvement of gait pattern was observed in 24% of all patients, headaches improved in 8%, and dizziness, mnestic disorder, and urinary incontinence each in 2% of cases (not shown graphically).

One of the 49 patients showed an infection of the VPS with skin flora (*Staphylococcus epidermidis*) after non-pathologic shuntography leading to subsequent explantation of the whole system. Thus, the complication rate of shuntography was 2%.

## Discussion

Diagnosing a shunt malfunctions is a challenge. The incidences of shunt obstructions after implantations of a VPS increase over time [31]. In contrast to previous studies, the present study identified obstruction of the VPS as a major mechanical complication in patients with normal pressure hydrocephalus. Subclinical infections of the CNS and as a result increased protein contents with higher viscosity might be a reason for obstruction of the shunts. Other causes could be material failures such as defective valves and subclinical minor peritoneal inflammations with occlusion of the distal catheter. All the more it was important to establish an elegant, minimally invasive, and safe method for diagnosing shunt dysfunctions in this patient population. In an experienced team of neurosurgeons and radiologists, shuntographies take only about 15 min in clinical practice. The low infection rate and the straight-forward information in cases of obstructed or disconnected VPS profile speak in favor of contrast-enhanced shuntography. The high level of reliability of contrast-enhanced shuntography suggests that this simple method provides clinically reliable information about the patency of shunt systems [30]. Neurosurgical follow-up is essential to determine if and why a patient’s clinical condition deteriorates after shunt placement in patients with iNPH [11]. According to the present study in almost every fourth patient a pathological shuntogram with signs of a mechanical complication can be anticipated. This result is of great importance for strict follow-up and management of these patients who always need surgical therapy [3]. So far contrast-enhanced shuntography is standard of care in every neurosurgical clinic. Doctors might avoid to use this method because of their lack of experience. If mechanical shunt dysfunction is suspected, the VPS can be visualized on plain radiographs to exclude disconnections; obstructions can therefore not be detected. The most common consequence of such diagnostics is the replacement of the complete VPS. Shuntography also allows selective visualization of the location of a potential mechanical problem in the path of the VPS. This is a major advantage of this diagnostic procedure, since it is not always necessary to replace an entire VPS system, but only its “defective” part. The present work showed that about 60% of the patients would be exposed to unnecessary revision surgeries and associated complications. Other studies evaluating unnecessarily revised VPS systems do not exist at this time. On average shunt infections occur in up to 8% of cases. Shunt infections are always a definitive reason for revision surgery [7, 22]. However, literature also reports infection rates of up to 12%, especially in combination of shunt exposure and intraoperative functional testing of the shunt in cases of suspected mechanical complications [6]. The latter approach is a widely used method. Contrast-enhanced shuntography however is a simple and effective method with a low-infection rate (2% of cases) to assess the patency of VPS in patients with normal-pressure hydrocephalus and suspected mechanical complications [2, 30]. Therefore, it seems advisable to carry out a contrast agent-assisted shuntography before any revision surgery in order to localize mechanical problems more accurately and thus reduce the duration of revision surgeries and postoperative infection rates. In contrast to an alternative method of using radionuclides for shuntography, contrast-enhanced shunt imaging shows better spatial–temporal resolution, allowing focused local surgical revision [4, 15, 20, 26, 30]. In our collective clinical outcome of those patients after revision surgery due to a pathological shuntogram and those patients after conservative therapy due to an inconspicuous shuntogram showed no significant difference. On the one hand, this could be attributed to the relatively small number of cases for sufficient statistical evaluation. On the other hand, surgical elimination of the obstruction in the shunt system is only one side of the coin. Complementary treatment of patients postoperatively (physiotherapy, educational measures, supportive medication) as well as close neurosurgical follow-up is probably just as important and the fundaments during exclusively conservative therapies in patients with persisting or recurrent symptoms after VPS placement.

## Limitations

The present study has certain limitations. Some patients’ records were incomplete which significantly reduced the number of included patients. In addition, the study includes patients with idiopathic normal pressure hydrocephalus which were treated with various valve types. Initial valve settings, onset and duration of symptoms, and the time between implantation of the VPS and shuntography are heterogenous in this retrospective cohort study.

## Conclusion

Contrast-enhanced shuntography is a safe and reliable clinical method for the diagnosis of mechanical shunt complications in patients with normal-pressure hydrocephalus. More than 50% of patients benefit from revision surgeries triggered by pathological shuntograms which identified the causes of mechanical shunt complications early and selectively.
